# Fungicides and strawberry pollination–Effects on floral scent, pollen attributes and bumblebee behavior

**DOI:** 10.1371/journal.pone.0289283

**Published:** 2023-07-27

**Authors:** Ann-Cathrin Voß, Madeleine Hauertmann, Michelle-Celine Laufer, Alexander Lach, Robert R. Junker, Elisabeth J. Eilers

**Affiliations:** 1 Department of Chemical Ecology, Bielefeld University, Bielefeld, Germany; 2 Evolutionary Ecology of Plants, Philipps-University Marburg, Marburg, Germany; University of Carthage, TUNISIA

## Abstract

Fungicides are used in agriculture to protect crops from various fungal diseases. However, they may modulate the plants metabolism. Moreover, fungicides can accumulate in the environment and may cause toxic effects on non-target organisms such as nectar microbes and pollinators. Nectar microbes contribute to the volatile profile of flowers and can influence pollinators behaviour. Thus, fungicide treatment could potentially affect the pollination. In this study, we investigated the influence of fungicide treatment on floral attributes as well as the behavioural impact on bumblebees. In separate experiments, we used one or both strawberry cultivars (*Fragaria* × *ananassa* var. Darselect and Malwina), which were either kept untreated (control) or treated with either Cuprozin^®^ progress or SWITCH^®^ fungicide. We analysed various flower traits including volatiles, pollen weight, pollen protein, and the attraction of bumblebees towards the flowers in the greenhouse. Additionally, we analysed the viability of pollen and pollen live-to-dead ratio, as well as the composition of nectar fungi in the field. A treatment with Cuprozin^®^ progress led to a lower emission of floral volatiles and a slightly lower pollen protein content. This had no impact on the visit latency of bumblebees but on the overall visit frequency of these flowers. The treatment with the fungicide SWITCH^®^ resulted in a higher emission of floral volatiles as well as a delayed first visit by bumblebees. Furthermore, flowers of control plants were visited more often than those treated with the two fungicides. Plant-pollinator interactions are highly complex, with many contributing factors. Fungicides can have an impact on the pollen quality and pollinator attraction, potentially leading to an altered pollen dispersal by pollinators and a change in fruit quality.

## Introduction

To protect crops from various pests and diseases including fungi, numerous pesticides such as fungicides are used in agriculture [1]. However, many pesticides can be harmful to the environment, by accumulating in ecosystems and by having toxic effects on non-target organisms [1, 2]. To prevent fungal infections of commercially traded fruits, fungicides are often applied on open flowers worldwide, which means that pollinating insects come into direct contact with these fungicides [3–5]. Although most of the approved fungicides are considered non-toxic to pollinators, fungicide exposure can result, for example, in a reduced number of bumblebee workers, smaller queens, decreased survival and increased susceptibility to other pesticides [3, 6]. In addition, many of the active ingredients in fungicides can cause measurable changes not only in the target’s environment but also in the metabolism of the treated plants [7–9]. So far, there are only a few studies on the possible effects of agrochemicals on plant metabolism and even less is known about whether there are consequences for interactions between plants and animals, such as pollinating insects.

For pollinators, floral colour, visual pattern, and particularly the floral scent, act attractive [10]. Flowers emit various volatiles, such as terpenoids, benzenoids and phenylpropanoids [11–13]. These floral volatiles are not only derived from the primary and specialised metabolism of the plant [11] but can also be modified by nectar microbes that alter the nectar chemistry, such as the composition of sugars and amino acids [14–16]. Depending on the microbe species, effects on pollinators can differ. For example, artificial nectar with yeasts was visited more often and bumblebees removed more nectar compared to artificial nectar with less yeasts [14]. On the contrary, bacteria occurring in nectar at natural densities can cause avoidance by pollinators [17, 18]. By treating flowers with fungicides, the diversity and abundance of the microbial community of flowers may change, with consequences for the attractiveness of flowers towards pollinators [15]. Moreover, nectar yeasts thrive on pollen nutrients such as proteins and can thereby reduce the pollen quality and plant fecundity [16].

Most of the pollen protein originates from the cytoplasm and is only present in living pollen, thus, protein content and pollen viability highly correlate [19]. The application of fungicides during the flowering period may impair the nutrient uptake of the plants and, thus, pollen metabolism [20]. Consequently, pollination success could be affected, as bumblebees can distinguish between highly rewarding flowers, i.e., flowers with high pollen protein and nectar content, based on the scent and taste of pollen, increasing their foraging efficiency [19]. For adult female bumblebees and larvae, pollen protein is crucial, and its quality can impact their survival and oviposition [21, 22].

For some fruit crops, such as the cultivated strawberry (*Fragaria* × *ananassa* Duchesne, Rosaceae), a wide variety of fungicides are applied to prevent economic losses [23, 24]. The various strawberry cultivars differ in the total amount and composition of floral volatiles that attract pollinating insects [12, 25, 26]. Strawberry plants benefit from insect pollination because cross-fertilisation enhances the fruit quality and results in a higher seed number and lower fruit decay [27, 28]. Bumblebees (*Bombus* spp.) which are common pollinators in Europe, North America and Asia, have been deployed as pollinators in strawberry farming in these continents since the 1980s [10]. Bumblebees can distinguish flowers, varying in their floral traits such as differences in temperature, pollen amount or nectar sugar concentration and composition [14, 19]. Moreover, they can differentiate between volatile patterns of different strawberry cultivars, which only differ in the emitted quantity [29]. Thus, even small changes in floral traits induced by environmental challenges, such as pollutants, might affect the behaviour of pollinators.

This study aimed to investigate the impact of fungicide treatment on floral attributes and, thereby, the attractiveness of strawberry flowers for bumblebees. During the flowering time, plants of two strawberry cultivars (*F*. x *ananassa* var. Darselect and Malwina) were either kept untreated (control) or treated with the copper-based broad-spectrum fungicide / bactericide Cuprozin^®^ progress, which is also permitted in organic farming, or with the commonly used systemic fungicide SWITCH^®^, with the active ingredients cyprodinil and fludioxonil (safety data sheets, [30]). According to the safety data sheets, it is stated that both fungicides are not harmful to pollinators. Also, SWITCH^®^ mixed with other fungicides has been found to not alter pollinator behaviour [31, 32], but Cuprozin^®^ progress was found to reduce nectar removal by honeybees [33]. However, to the best of our knowledge, there are no studies regarding the impacts of these two fungicides with the focus on bumblebee foraging behaviour. Considering that the two fungicides may change the plant metabolism and are detrimental to certain microbes, we expected that the treatments would affect the flower volatile profiles and the composition of fungal species in the nectar. Due to the impact of fungicides on the metabolism and the effect of nectar fungi on pollen, we also expected a lower pollen weight, live-to-dead ratio and pollen protein content. Moreover, we hypothesised that bumblebees are more attracted by control plants than fungicide-treated plants because of the altered volatile profiles. We defined the attractiveness of a plant as the sum of their visual and olfactory cues which lead to a higher visitation rate and in a field environment to a longer flower constancy of the pollinators [26]. Furthermore, the duration of the first visit was expected to be shorter on fungicide-treated plants than on control plants due to the differences in pollen quality. However, we expected the treatment effects on the frequency and duration of plant visits by bumblebees to vanish over the trial period. Finally, the frequency and duration of plant visits were expected to differ between the strawberry cultivars, as we showed previously that the same cultivars differ in metabolic composition and fruit size [9].

## Materials and methods

### Plant cultivation

Regardless of the year or environment all plants were planted in 4 L pots (15 x 15 x 23 cm) filled with a substrate mixture of 1:1 standard soil (Typ: P, Fruhstorfer Pikiererde, Hawita Group, Vechta, Germany) and river sand, which had been steamed at 95°C for 4 h. In addition, all plants were grown in a greenhouse chamber with a 16:8 h (light:dark) light regime and fluctuating temperature and humidity. Pots were randomly distributed on the greenhouse tables, watered *ad libitum* daily and plants fertilised once a week (Wuxal Professional Fertilizing, MANNA, Ammerbuch, Germany; NPK fertiliser solution 8-8-6 with trace nutrients: 8% total nitrogen, 8% diphosphorus pentoxide, 6% potassium oxide). An overview of all five experiments which were carried out in this study including their associated details such as year, environment or used cultivar can be find in the S1 Table.

To test the effect of fungicide treatment on the floral volatiles in experiment 1 (greenhouse n = 10; field n = 12), and on different pollen traits in experiment 3 (n = 12) and experiment 4 (n = 10), plants of *Fragaria* × *ananassa* cultivar Malwina (late cultivar, ripening time from mid June to mid July), were purchased from Kraege (Kraege Beerenpflanzen GmbH & Co.KG, Telgte, Germany) as pre-cultivated “frigo” plants in February 2020. Frigo plants are commonly used by farmers as they have a longer period of development before uprooting i.e., they have more roots and the flower primordia have already formed. After uprooting, frigo plants are then sorted and frozen from mid-November onwards [34]. Plants were planted in pots filled with a substrate and grown in a greenhouse chamber where pots were randomly distributed on the greenhouse tables, daily watered and plants fertilised once a week (see above). After four weeks of growth the first fungicide treatment took place (see below).

After the third fungicide treatment a subset of plants of each fungicide treatment group of experiment 1 (field; n = 12) and all plants of experiment 3 (pollen traits; n = 12) were transferred to a common garden field site nearby (latitude: 52.033684, longitude: 8.495052; 146 m a. s. l.) to capture a more natural floral volatile pattern (additional methods in S1 Method).

To test the effects of fungicides on the nectar fungi composition as a potential cause for alternations in floral volatile patterns [14, 15], in experiment 2 (n = 2–3) Malwina plants were analysed for the presence and composition of fungal species in nectar. Therefore, in February 2021, Malwina plants were acquired as frigo plants from erdbeerprofi.de (Erdbeerprofi GmbH, Landsberg, Germany) and were planted in 4 L pots on 19. April 2021. Malwina plants were chosen as the bumblebees showed a more distinct response with this cultivar during the trials than with Darselect plants. After eight weeks of growth in the greenhouse (14. June 2021) as well as after the third fungicide treatment (see below), plants of each fungicide treatment were transferred to the common garden field site nearby to enable infestation of the floral nectar with naturally occurring microbes (additional methods in S1 Method).

For the bumblebee trials in experiment 5 (n = 19–22), two *Fragaria* × *ananassa* cultivars, Darselect (early cultivar, fruit ripening from end-May to mid-June) and Malwina, were purchased as frigo plants from Kraege in February 2020. An early (Darselect) and a late (Malwina) cultivar were chosen to elongate the time frame for the different experiments as well as to find cultivar-specific responses of the bumblebee in the behavioural trials. To extend the availability of flowers during the trials, plants were set-up consecutively in three batches, planted on 06. March 2020, 20. March 2020 and 03. April 2020. Replicates of Darselect were equally distributed among the first and second batch, and Malwina plants among the second and third batch. Plants of both cultivars were planted in substrate filled pots and grown in a greenhouse chamber with pots randomly distributed on greenhouse tables, watered daily and fertilised once a week (see above). The first fungicide treatment (see below) was applied after four weeks of growth.

### Fungicide treatments

Plants from all experiments were randomly divided into three groups and assigned to one of three fungicide treatments. One control group (CTR) was kept untreated i.e., without any application. Plants of the second group were treated with Cuprozin^®^ progress (CU; 383 g/L; Spiess-Urania Chemicals GmbH, Hamburg, Germany), a contact fungicide and bactericide with copper hydroxide as the active ingredient. Plants of the third group were treated with SWITCH^®^, a co-formulation of the systemic fungicides cyprodinil and fludioxonil [FR (fruit rot); 2 g/L; Syngenta Agro GmbH, Basel, Switzerland], which act on the methionine biosynthesis and the histidine-kinase involved in osmotic signal transduction of fungi [30]. CU was applied three times during the season (once before, twice during flowering) with 900 ml of the solution on the plants to achieve a rate of approx. 10.53 mg copper hydroxide per plant per application time point in an interval of 7–8 days. FR was applied twice (once before and once during flowering) with 250 ml of the solution on the plants to achieve a rate of approx. 4.69 mg cyprodinil and 3.13 mg fludioxonil per plant per time point in an interval of 16 days. The fungicides were applied with separate pressure sprayers, following the supplier’s recommendations for the water application rates and intervals for farmers. For application, plants were transferred to an adjacent greenhouse chamber and remained there for 24 h before placing them back in the original greenhouse chamber.

### Experiment 1: Collection of floral volatile organic compounds and chemical analysis

The floral volatile organic compounds were collected from Malwina plants in the greenhouse (7–8 plants per treatment, 2020) and the field (9–11 plants per treatment, 2020) following the method of Ulrich and Olbricht [25] and Kallenbach et al. [35]. The volatiles were collected from both the greenhouse-grown and the field-grown plants to link the volatile data to the bumblebee behaviour data and to show that the composition of the volatiles at both locations are similar. Polydimethylsiloxane (PDMS) tubes (Carl Roth GmbH + Co. KG, Karlsruhe, Germany) were cut into 0.5 cm pieces, soaked in 4:1 (v:v) acetonitrile: methanol for 3 h at 80°C and afterwards conditioned in a thermal desorption unit (TD; TD-30, Shimadzu, Kyoto, Japan) at 230°C for 30 min with a 60 ml min^-1^ flow. Polypropylene cups (80 ml, Pro-Pac, Vechta, Germany) were provided with two air holes of about 1 cm^2^ to ensure air circulation and release of condensing water, were attached to wooden sticks to the height of the flowers. For the volatile collection, one flower per plant was placed inside the cup with two conditioned PDMS tube pieces near the flower and volatiles were collected over an 8 h absorption period. As controls, volatiles were likewise collected in cups placed in close vicinity without a flower, to identify possible contaminants from surrounding volatiles. After volatile collection, the PDMS tubes were transferred to 2 ml glass vials and stored at -80°C until analysis. Additionally, the floral and receptacle diameter was measured per sampled flower.

The sample PDMS pieces and control PDMS pieces were thermally desorbed and analysed by gas chromatography mass spectrometry (TD-GC-MS; GC 2010plus–MS QP2020, Shimadzu, Kyoto, Japan) on a VF5-MS column (30 m × 0.2 mm ID, 10 m guard column, Varian, Palo Alto, CA, USA) with helium as carrier gas. The PDMS sample desorption was carried out at 230°C with a 60 ml min^-1^ flow and VOCs were cryo-trapped on Tenax^®^ at -20°C for 8 min. From the cryo-trap, volatiles were re-desorbed at 250°C for 3 min and transferred to the GC at 250°C in a 1:1 split mode. The GC column oven temperature program started at 50°C, was held for 5 min, increased by 10°C/min to 250°C, further increased by 30°C/min to 280°C and was finally held for 2 min. The MS ion source temperature was 230°C with an interface temperature of 250°C and a detector voltage of 0.5 kV. The MS scanned from minute 3.5 to minute 28.5 with a scan rate of 0.4 s/full scan, and the line spectra (range: 30–400 *m*/*z*) of the compounds were acquired in quadrupole MS mode. In addition to the samples, a standard alkane mixture (C7-C40, Sigma-Aldrich, Chemie GmbH, Munich, Germany) and 1-bromodecane (Sigma-Aldrich) diluted in n-heptane (CHEMSOLUTE, Th. Geyer GmbH & Co. KG, Renningen, Germany) was measured using the same method to calculate the retention indices [36]. The GC-MS Postrun analysis program (version 4.45, Shimadzu) was used to analyse the chromatograms. Mass spectra and retention indices of volatiles were compared to the NIST-database (NIST14, National Institute of Standards and Technology, Gaithersburg, Maryland, USA), the FFNSC3-database (University of Messina, Messina, Italy) specifically for fragrance analysis, the PubChem database [37] and Adams [38] for putative identification. Peaks were integrated based on the extracted ion chromatogram, and respective peaks occurring in control samples were subtracted. Contaminants of non-plant origin were excluded from further data processing, resulting in a list of typical floral fragrances based on the Pherobase database [39]. Furthermore, only compounds were kept in the dataset if the mean MS-signal intensity in at least one treatment group was higher than twice the mean intensity in the control samples, to exclude non-strawberry plant volatiles from surrounding plants in the field. Furthermore, volatiles that were present in at least 50% of the samples in at least one of the treatment groups were kept (similar as in [40]).

### Experiment 2: Analysis of floral nectar fungi

To collect nectar, glass capillaries (borosilicate glass capillary, 1 mm outer diameter, 0.139 mm wall thickness; Hilgenberg GmbH, Malsfeld, Germany) were heat-divided into two capillaries with very fine tips using a micropipette puller (model P-97, Sutter Instrument^®^, Novato, USA) (heat: 530°C, pull: 70, velocity: 70, time: 250). The nectar harvesting equipment was autoclaved before use. Nectar samples were collected according to Morrant et al. [41] from one open flower per plant of three CTR and CU, and four FR Malwina plants in the field. To collect the nectar of a strawberry flower, 50 μl of a 2% sterile sugar solution (to prevent the cells from bursting) was carefully pipetted onto the flower, incubated for 10 min, and subsequently reabsorbed with the fine glass capillary (22–30 μl). The filled capillary was transferred to a Zymo kit (ZymoBIOMICS™ 96 DNA Kit; Zymo Research EUROPE GMBH, Freiburg, Germany), shaken, stored on ice, and homogenised. Samples were frozen and stored at -80°C until analysis.

Samples were analysed by next-generation ITS2-amplicon sequencing using the primers ITS2 5′-GCATCGATGAAGAACGCAGC-3′ and R 5′-TCCTCCGCTTATTGATATGC-3′ [42] and microbiome profiling of isolated DNA was performed on the Qiita web platform [43] on which fungal amplicon sequence variants (ASVs) were obtained using DADA2 [44]. Prior to the statistical analysis of microbial communities, we performed a cumulative sum scaling (CSS) normalization (R package *metagenomeSeq* v1.28.2, [45]) on the count data to account for differences in sequencing depth among samples.

### Experiment 3: Pollen collection and measurements of viability

Pollen samples were taken from one flower per plant of 10–12 plants per treatment, from Malwina plants placed in the field (2020). The five anthers of each flower were weighed and placed in a reaction vessel with five drops of an aceto-carmine-mannitol staining solution [46]. The staining solution was prepared with a 1% carmine (Acrōs Organics, Geel, Belgium) - 45% acetic acid solution (VWR International S.A.S., Fontenay-sous-Bois, France), which was mixed with a 0.7 M D-mannitol solution in a 1:5 (v:v) ratio [19, 47]. The samples were homogenised, and one drop of the pollen solution was pipetted onto a slide, sealed with a cover glass and fixed at the edges with nail polish (cosnova GmbH, Sulzbach am Taunus, Germany). The samples were scanned with a Mirax desk scanner (Carl Zeiss, Oberkochen, Germany) and the files analysed using the open-source software QuPath [48]. The dyed pollen were manually classified into three categories [alive (red), dead (yellow), unknown] in ten samples to generate data for a training data set. Afterwards, the recognition algorithm was trained with this data. These two steps were repeated until the algorithm could error-free identify the categories. At the end the number of pollen from the three categories and the total number of pollen in each sample were counted by the trained software and subsequently exported. The live-to-dead ratio of pollen grains was calculated as a proxy to estimate the variation in pollen quality between the cultivars.

### Experiment 4: Measurements of pollen weight and protein content

To determine the pollen weight and protein content, separate anthers were sampled from one flower per plant of 9–10 plants per treatment, from Malwina plants grown in the greenhouse (2020). Pollen was weighed, oven-dried at 36°C for at least 24 h and subsequently weighed to determine the dry weight of the pollen. The protein content was determined using a method modified after Bradford [49] and Eilers et al., [50]. For each sample, 1 mg dried pollen was homogenised with 4°C cold 500 μl of 0.1 mol/L NaOH. Another 500 μl 0.1 mol/L NaOH was added, the sample sonicated for 10 min, and centrifuged. Supernatants were measured on a microplate photometer (Multiskan Go, Thermo Scientific, Waltham, MA, USA) with Bradford solution (Panreac AppliChem, Darmstadt, Germany) at room temperature at 595 nm, using a calibration curve of bovine serum albumin (Carl Roth GmbH + Co. KG, Karlsruhe, Germany).

### Experiment 5: Behavioural observations of bumblebees

Three small bumblebee colonies (*B*. *terrestris*) each with a queen, several workers, and their brood, were purchased (Biobest Group NV, Westerlo, Belgium; distributed via Katz Biotech AG, Baruth, Germany) in 2020. Each bumblebee nest was kept in a plastic box (23 x 35 x 30 cm, perforated side walls, box filled with organic cosmetic cotton) equipped with a landing ramp on the entrance / exit (round hole of 3 cm diameter) and a sliding gate to control the entry and exit of the bumblebees during the trials. The three colony boxes were colour coded (red, white, and green colony) to later match the bumblebees to their original nest. The boxes were placed on a wooden supporting construction of in 30 cm height in a rectangular net-tent (H x L x B: 2 x 4.3 x 1.4 m) inside a greenhouse chamber. The bumblebees were provided with ground pollen bread (Buxtrade, Buxtehude, Germany) and a 40% sugar-water solution containing one spoon of honey L^-1^. The food sources were placed on an orange plastic sheet (approx. 1 m^2^, taped to the floor) on the opposite side of the wooden supporting construction in the tent to establish a feeding side. Two weeks after colony arrival, bumblebees were conditioned by removing the food sources and exchanging them with six flowering plants, one plant of each treatment and cultivar (exclusively placed on the orange sheet) to train the bumblebees to forage pollen and nectar from strawberry flowers.

The first behavioural trials were performed at least three days after the last fungicide application. The night before an experimental day, the sliding gates were closed, and the remaining bumblebees outside the nest were transferred back to their original nest. Observations were performed in the morning (7:30 am to 1:30 pm) to reduce stress and allow free-flight behaviour for the rest of the day. Bumblebees were separately offered a plant set of one plant of each treatment group of one cultivar, positioned in a triangle on the orange sheet. The position was rearranged after each bumblebee, and the plant set was exchanged after three bumblebees. All bumblebees started from the same position out of a Flaubert tube (APIFORMES, Stuttgart, Germany) near the nest with approx. 2.5 m distance to the plant set and were recorded for 15 min. Preliminary tests revealed an optimal test duration of 15 min, as several bumblebees took up to 10 min to approach the plants. Bumblebee behaviour was recorded atop of the orange sheet with a camera (Interchangeable lens digital camera A7 III Sony, Tokyo, Japan; Lens: FE 28–70 mm F3.5–5.6 OSS; resolution: 1080 p; display aspect ratio: 16:9; bit rate: 60.0 mb/s; frame rate: 50 FPS). The focus and brightness of the camera were adjusted for each observation before the recording. In addition, information about the test- and plant IDs as well as the environmental conditions (temperature, humidity, and time), were filmed. Furthermore, the plant position in the triangle and the status of the flower (closed, open, decayed) and fruits (unripe, ripe) were recorded.

After each trial, the bumblebee was recaptured and marked (Uni Posca Marker; Mitsubishi Pencil Co., Ltd., Tokyo, Japan) with the respective colony colour to avoid double testing. The spot remained on the bumblebees back until the end of all observation trials. Until all trials of the day were conducted, the tested bumblebees were placed in a net tent (H x L x B: 60 x 60 x 60 cm) and were provided with pollen bread and sugar-water. Overall, in this study, 34 plant triplets, 15 Darselect and 19 Malwina, were tested with plants from the climate chamber, which amounted to 102 observed bumblebees.

Prior to the video analyses, the additional information about the treatment was deleted from the video and file name to avoid personal bias, and videos were analysed using BORIS, version 7.9.8.– 2020–01–28 [51]. The latency until the first flower visit, the length of the first flower visit, as well as the overall plant visit frequencies and durations of each bumblebee were determined.

### Statistical analyses

The statistical analyses and data visualisation were performed using RStudio [52] in R 4.1.2 [53]. All figures were generated using the package *plyr* [54]. In general, all linear models (LM) were performed for response factors with a normal error distribution and generalised linear models GLM) or generalised linear mixed-effects models (GLMM) were performed for responses with a Gamma distribution using the package *lme4* [55]. Model variance homogeneity and normal distribution of residuals were checked by visual inspection [56]. Through the marginality rule, model simplifications based on chi-squared likelihood ratio tests for (G)LMM were performed using the dropterm function (package *MASS* [57]) to generate p-values for single factors and factor interactions (R package *car* [58]) as well as the coef function for orthogonal contrasts between the groups of a factor (R package *stats* [53]). If necessary, the data were shifted by 1e^-07^ along the x-axis to positive x-values to fit a Gamma distribution. In separate (G)LMs (gaussian, link: identity or log; Gamma, link: inverse), the total floral diameter, the receptacle diameter, and the amount of each of the 13 volatiles were used as response factors to test the effect of the fungicide treatment (CTR, CU, FR) serving as explanatory variable. The floral volatile composition of greenhouse and field plants was visualised in separate non-metric multidimensional scaling (NMDS) with Wisconsin double standardisation of square-root transformed data, using the Kulczynski distance (package *vegan* [59]). Concomitantly, permutational multivariate analyses of variance using distance matrices (ADONIS) based on Kulczynski distances were used to test the effects of the factor fungicide treatment (CTR, CU, FR) on volatile composition.

To investigate the impact of the fungicides on the occurrence and composition of nectar fungi, only amplicon sequence variants (ASVs) linked to the taxonomic domain of the fungi were used. The rarefaction of the non-normalized data and the evaluation of the Shannon diversity were performed with the package *rtk* [60]. Due to the low sample size, no statistical analysis of the effects of fungicide treatment on the composition of the nectar fungi was possible.

The influence of the fungicide treatment on pollen dry weight (dw), protein content, viable pollen, total number of pollen grains and live-to-dead ratio of pollen grains of Malwina plants were analysed with GLMs (Gamma or poisson, link: inverse or log) and subsequently with a Tukey *post-hoc* test.

To analyse the effects of plant treatment on pollinators, the response factors latency until the first flower visit (Gamma, link: log), duration of first visit (Gamma, link: log), frequency of plant visits (poisson, link: log) and total duration of plant visits (Gamma, link: log) of bumblebees were examined in four separate GLMMs. In these models, we initially included fungicide treatment as the factorial predictor, cultivar (Darselect, Malwina), number of open flowers and number of ripe fruits as fixed effects, and the plant position and colony IDs as a random effects. After model simplification the final models, included fungicide treatment as the factorial predictor, cultivar (Darselect, Malwina) and number of open flowers as fixed effects, and the plant position and in one case colony ID as a random effect. The factor cultivar was only included as additive, not as interaction, as the two cultivars (Darselect and Malwina) were considered independent.

## Results

### Differences in floral traits due to fungicide treatment

**Fig 1:**
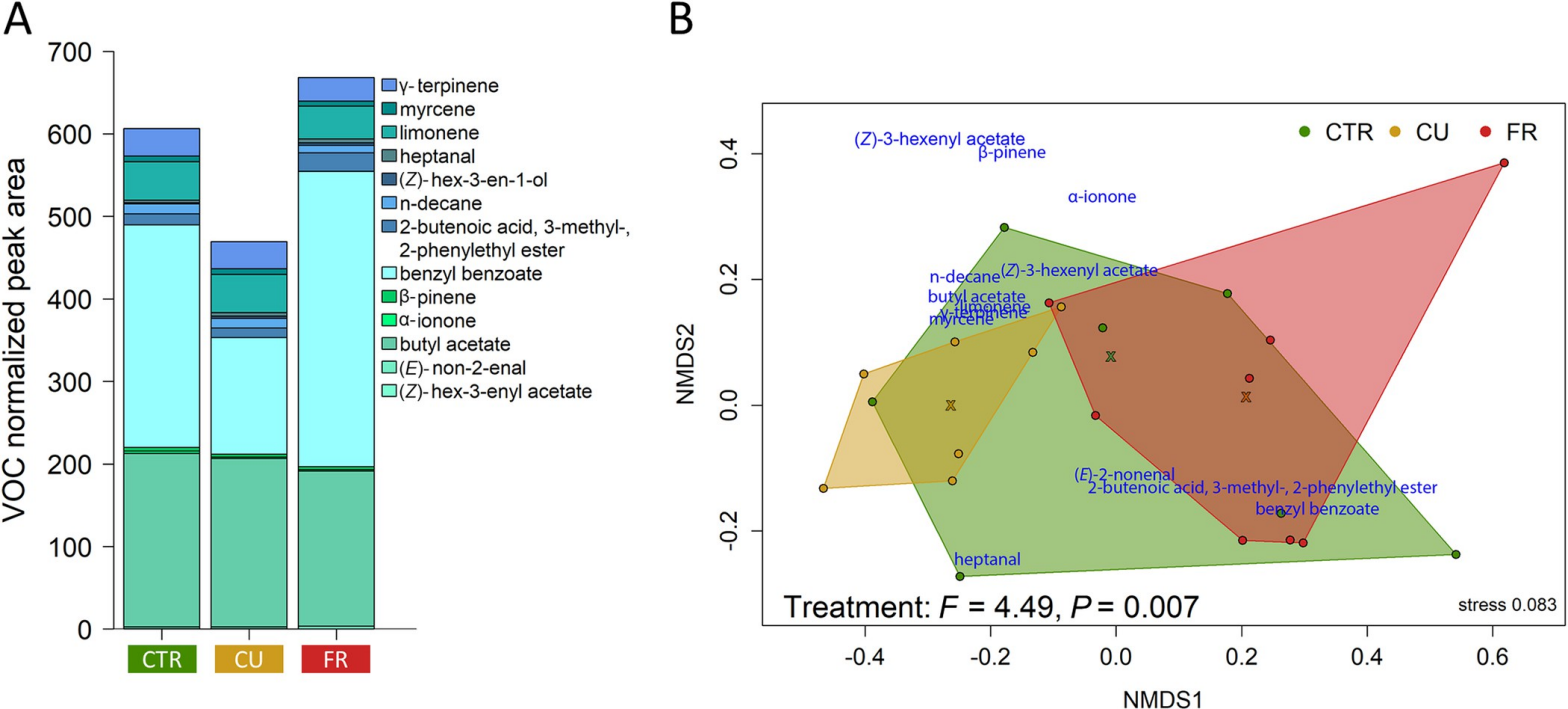
Flower volatiles of fungicide treated strawberry plants grown in the greenhouse (2020). Plants (*Fragaria × ananassa* var. Malwina) were untreated [control (CTR)] or fungicide-treated [Cuprozin^®^ progress (CU), SWITCH^®^ (FR)]. (**A**) The volatile composition (averaged over replicates within groups) and (**B**) non-metric multidimensional scaling (NMDS; with Kulczinsnky distance matrix) of the volatile composition with scores (coloured symbols; samples within each group are surrounded by convex hulls and the corresponding medians of the groups shown as crosses) and loadings (blue compound names). Results of the ADONIS are shown in the graph (**B**); n = 7–8 replicates per fungicide treatment.

In total, 13 floral volatiles, of which one alcohol and alkane, two aldehydes, four esters and five terpenes were detected in the floral headspace of plants grown in the greenhouse (May 2020, S2 Table). The contents of only two of these compounds, benzyl benzoate and 2-butenoic acid, 3-methyl-, 2-phenylethyl ester, differed significantly in the fungicide treatment (Fig 1A, S3 Table). The content of benzyl benzoate was significantly lower in CU-treated flowers compared to CTR and FR-treated flowers (Fig 1A), whereas the content of 2-butenoic acid and 3-methyl-, 2-phenylethyl ester was significantly lower in CU-treated and by trend lower in control flowers compared to FR-treated plants (Fig 1A). The total normalised peak area of the floral volatiles (sum of 13 compounds) was not significantly different between plants of the different fungicide treatments (Fig 1A, S3 Table). Despite a large overlap of floral volatile data in an NMDS analysis, significant differences were found between the profiles of the plants exposed to the three different fungicide treatments (Fig 1B). In general, the total floral diameter and receptacle diameter of the sampled flowers of the cultivar Malwina, grown in the greenhouse (2020), ranged between 15.4–27.6 mm and 4.4–6.3 mm, respectively. Both traits were not significantly affected by the fungicide treatment (floral diameter: *χ^2^* = 1.95, *P* = 0.378; receptacle diameter: *χ^2^* = 0.8, *P* = 0.671, N = one flower per plant from 6–9 plants per treatment).

In the floral headspace of Malwina strawberry plants placed in the field in May 2020, the compound 2-butenoic acid, 3-methyl-, 2-phenylethyl ester was only present in one sample, and α-ionone could not be detected at all (S4 Table). Neither the total amount nor the individual compounds of the field-collected flower volatiles were shown to be affected in their amount by the fungicide treatment (S2A Fig and S4 Table). The profiles of the floral volatiles of the plants of the different treatments overlapped largely in an NMDS but were not significantly affected by the treatment (S2B Fig).

### Occurrence of strawberry nectar fungi after fungicide treatment

**Fig 2:**
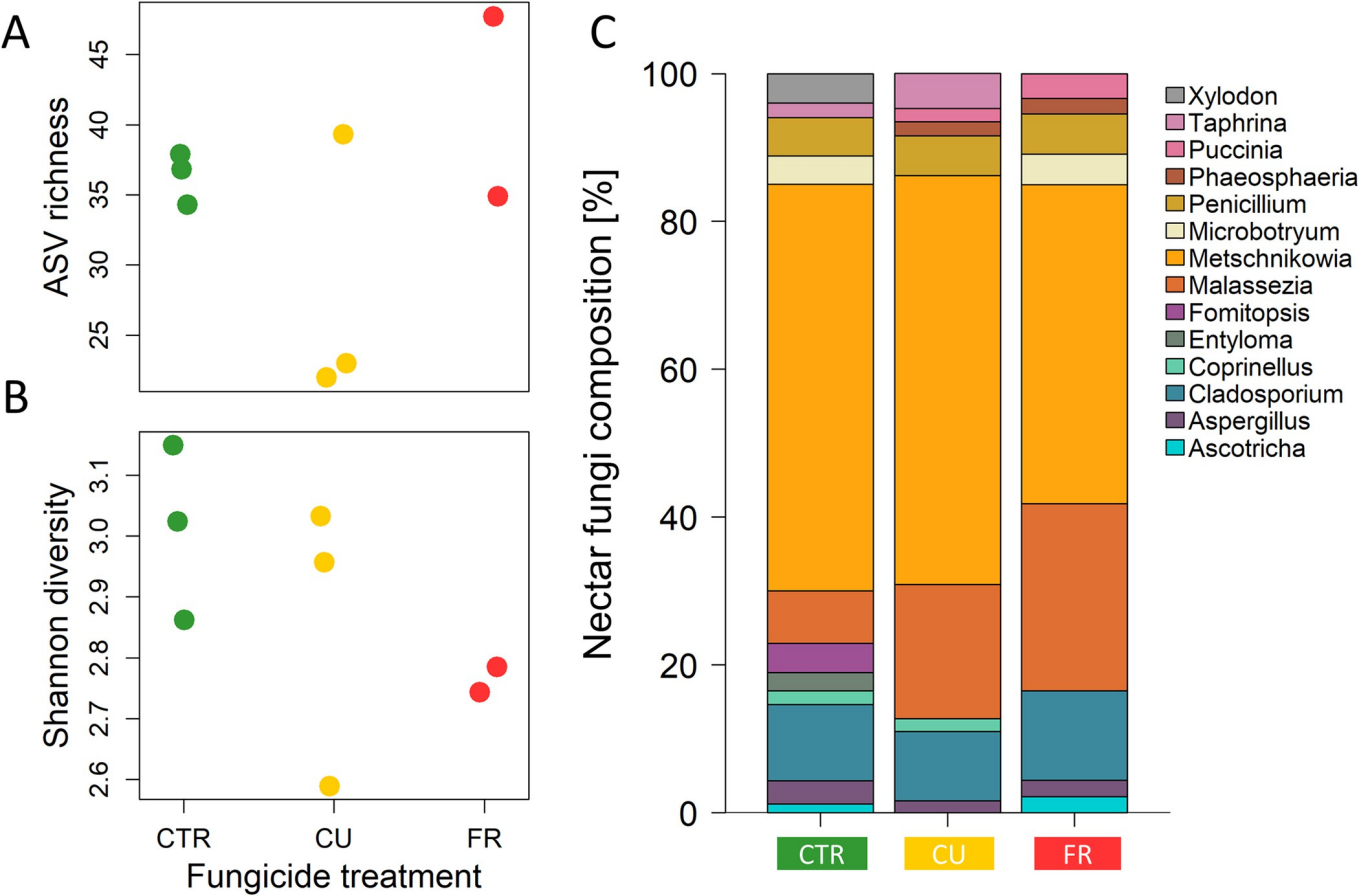
Amplicon sequence variants (ASV) of nectar fungi of fungicide treated plants placed in the field (2021). Displayed are the richness (**A**), Shannon diversity (**B**) and the composition (**C**) of nectar fungi of flowers from control (CTR; green) or fungicide-treated [Cuprozin^®^ progress (CU; yellow), SWITCH^®^ (FR; red)] plants (*Fragaria* × *ananassa* var. Malwina). Data points of the richness (**A**) and Shannon diversity (**B**) is presented as strip plot while the composition (**C**) is presented in a stacked bar plot which represents the mean distribution in ASV over the fungicide treatment groups. Presented are the fungi genera with an abundance of more than 5% per sample; n = 2–3 replicates per fungicide treatment.

Overall, the ITS2-amplicon sequencing of nectar samples in the field in June 2021 resulted, accumulated over all samples, in 605 distinct ASVs. The taxonomic classification of the fungi domain revealed 129 fungal species from the phyla Ascomycota and Basidiomycota, including 16 classes and 42 orders, as well as 82 families and 113 genera. ASV richness (number of species) was seemingly higher in the nectar of FR-treated flowers (2 replicates) and eventually lower in the nectar of CU-treated flowers (3 replicates) compared to the nectar of control flowers (3 replicates) (Fig 2A). The Shannon diversity was similar in all three treatment groups (2–3 replicates per treatment). However, the control group showed a slightly higher value than the fungicide groups, with the FR group being lower than the CU group (Fig 2B).

After filtering, i.e., removing ASVs that occur in an abundance of < 5% across all samples, 10 fungal classes, 13 orders and families and 14 genera were retained in the data set (Fig 2C). About half of the fungal species in the nectar belonged to the genus *Metschnikowia* (45–55%), followed by the genera *Malassezia* (7.1–25.3%) and *Cladosporium* (9.3–12.1%) (Fig 2C). The other genera were represented by around or less than 5% (Fig 2C). *Entyloma*, *Fomitopsis* and *Xylodon* species were only found in the nectar of flowers of control plants, while *Phaeosphaeria* and *Puccinia* were only present in the nectar of flowers of fungicide-treated plants (Fig 2C). No *Microbotryum* and *Ascotricha* species were found in the nectar of CU treatment flowers, whereas in the nectar of FR treatment flowers, none of the *Coprinellus* and *Taphrina* species were found (Fig 2C).

### Impact of fungicide treatment on pollen traits

The dry weight of the pollen collected from strawberry plants of the cultivar Malwina, grown in the greenhouse in 2020, was on average, 2.3 mg in flowers of CTR and CU-treated plants, while those of FR-treated plants had a slightly lower weight with 1.9 mg. However, the differences were not significantly different (Table 1). Overall, the pollen protein content was relatively low in the plants. Pollen of FR-treated plants had a higher protein content (3.04%) than those of CTR plants (2.2%), while pollen of CU-treated plants had the lowest protein content (0.68%). Regardless of these differences, the protein content was not influenced by the fungicide treatment. Although the variation in the protein content of FR-treated plants (*χ^2^* = 5.75, *P* = 0.05) was significantly smaller than in pollen of control plants (Table 1).

**Table 1:** Statistical outcome for pollen traits of fungicide treated plants.

response factor	fixed effect	parameter estimates ± s.e.		*P* value	N plants	*χ^2^*	statistical test
pollen dw	treatment (CTR)	CU	0.01 ± 0.07	0.875	9	2.01	GLM (Gamma, log link)
		FR	0.09 ± 0.07	0.202	10		
protein content	treatment (CTR)	CU	1.02 ± 0.93	0.279	9	2.98	GLM (Gamma, inverse link)
		FR	-0.13 ± 0.32	0.697	10		
viable pollen	treatment (CTR)	CU	0.01 ± 0	**0.017**	10	14.26	GLM (Gamma, inverse link)
		FR	0.001 ± 0	0.465	12		

Pollen dry weight (dw) and protein content (greenhouse, 2020) as well as number of viable pollen (field, 2020) from control (CTR) or fungicide-treated [Cuprozin^®^ progress (CU), SWITCH^®^ (FR)] plants (*Fragaria* × *ananassa* var. Malwina). Displayed are parameter estimates with std. errors and *P*-values indicating levels of significance obtained from generalised linear models (GLM) for the effect of the treatment (CTR, CU, FR). The number of replicates is presented as well as the *χ^2^* value for fixed effects.

**Fig 3:**
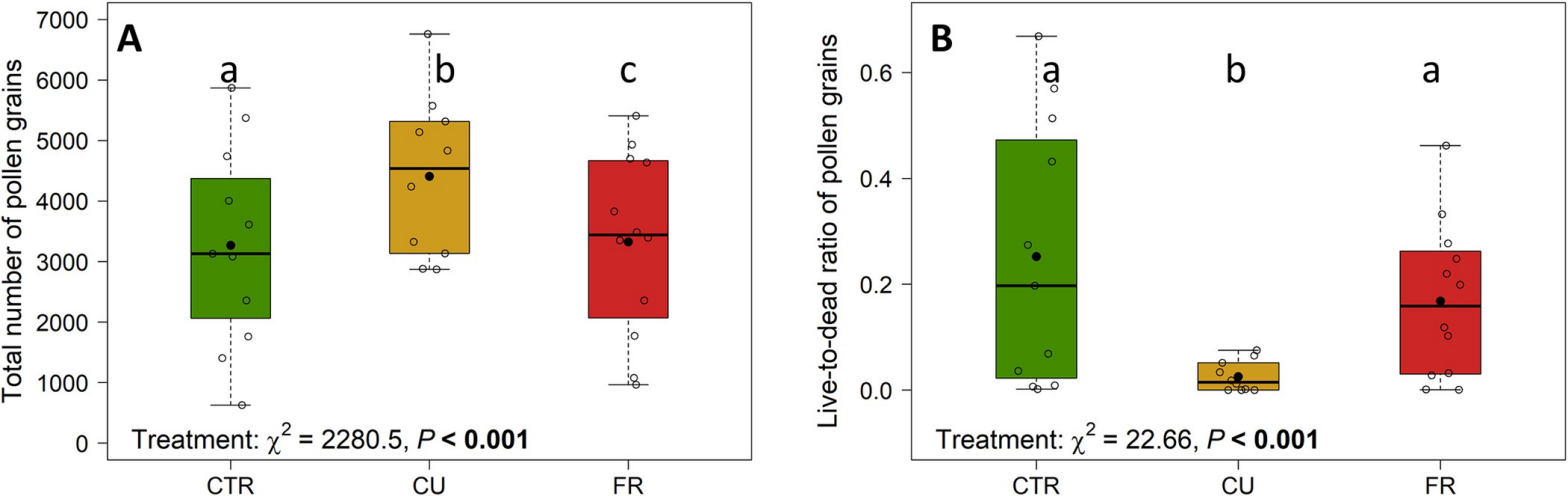
Pollen traits of fungicide treated strawberry plants placed in the field (2020). Total number of pollen grains (**A**) and live-to-dead ratio of pollen grains (**B**) of pollen from control (CTR; green) or fungicide-treated [Cuprozin^®^ progress (CU; yellow), SWITCH^®^ (FR; red)] plants (*Fragaria* × *ananassa* var. Malwina). Data is presented as box-whisker plots with interquartile ranges (IQR; boxes) including medians (horizontal lines) and whiskers (extending to the most extreme data points with a maximum of 1.5 times the IQR), while black dots indicate the means; individual values are given as circles. Significant values (P < 0.05) of the generalised linear models are highlighted in bold and the Tukey post hoc test is indicated by different lowercase letters; n = 10–12 replicates per fungicide treatment.

The fungicide treatment significantly influenced the total number of pollen grains of flowers in the field (2020), with CU- and FR-treated plants having a 35% and 2% higher number of grains than CTR plants (Fig 3A). The live-to-dead ratio of pollen in flowers of Malwina plants growing in the field was significantly affected by the fungicide treatment (Fig 3B). Flowers of the CTR and FR treatment had a significant higher live-to-dead ratio than those of the CU-treated plants (Fig 3B). The number of viable pollen mirrored this pattern (Table 1).

### Behavioural responses of bumblebees towards fungicide treated plants

**Fig 4:**
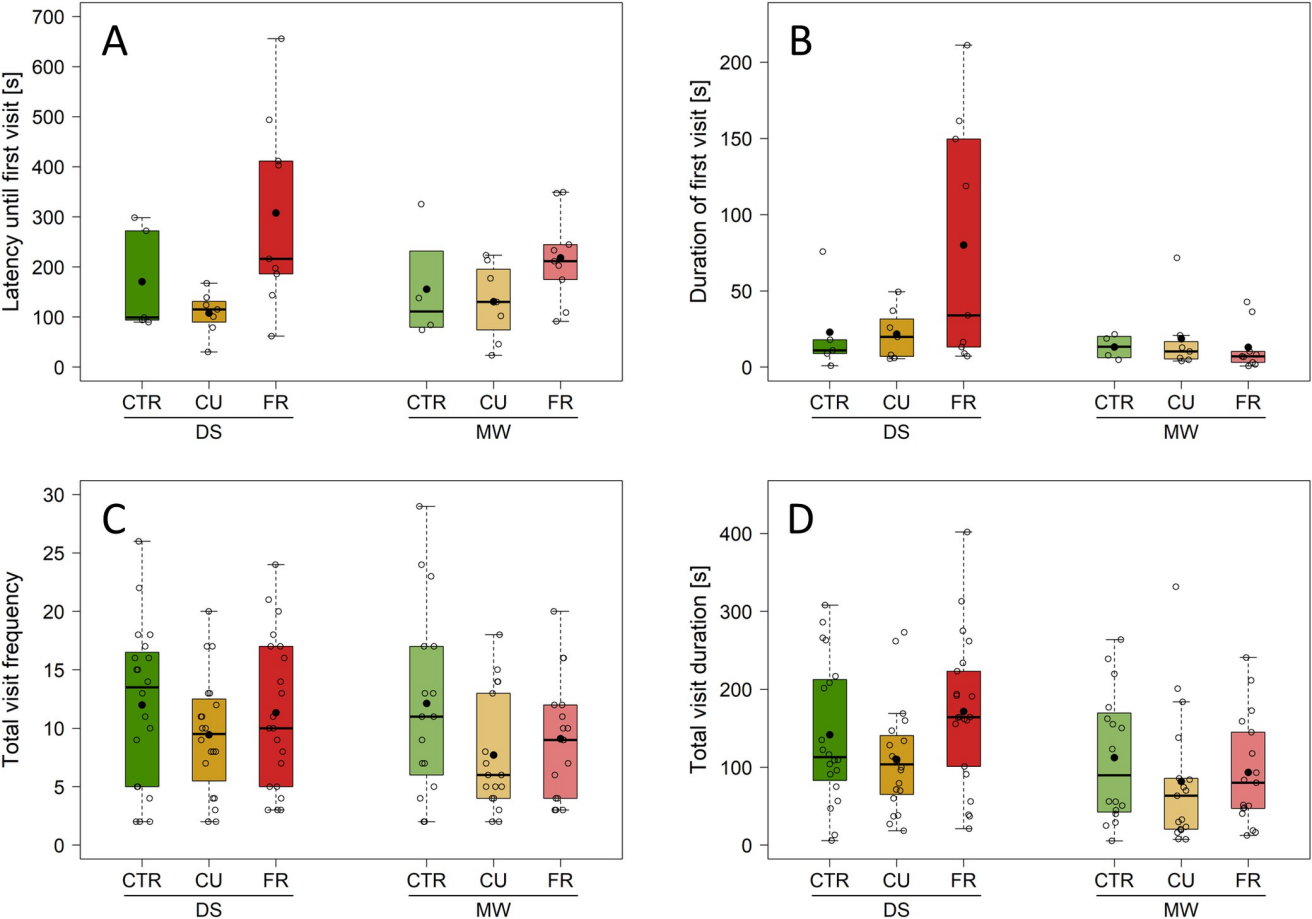
Behavioural responses of *Bombus terrestris* workers towards treated flowers (2020). Plants [*Fragaria* × *ananassa* var. Darselect (DS), Malwina (MW)], grown and offered in a greenhouse were untreated [control (CTR)] or fungicide-treated [Cuprozin^®^ progress (CU), SWITCH^®^ (FR)]. Displayed are the latency until the first flower visit (**A**), duration of that first visit (**B**), frequency of plant visits (**C**) and total duration of plant visits (**D**). Data is presented as box-whisker plots with interquartile ranges (IQR; boxes) including medians (horizontal lines) and whiskers (extending to the most extreme data points with a maximum of 1.5 times the IQR), while black dots indicate the means; individual values are given as circles. Significant differences are shown in Table 2; **A, B**: n = 4–9, **C, D**: n = 40–56 replicates per fungicide treatment.

Flowers of FR-treated greenhouse plants (2020) were visited significantly later (262.93 ± 153.27 s) than those of the CU or CTR treatment (163.92 ± 103.37 s and 119.41 ± 62.49 s respectively), while there was no significant effect of cultivar regarding the latency until the first flower visit (Fig 4A, Table 2). Not significant, but with a tendency towards significance, the duration of this first visit was 2.5 times and 2.3 times longer on flowers of FR-treated than on CTR and CU-treated plants, respectively. During this first visit, flowers of Malwina plants were visited for a significantly shorter time than flowers of Darselect plants (Fig 4B, Table 2).

**Table 2:** Statistical outcome of the behavioural responds of *Bombus terrestris* towards treated flowers.

Response factor	fixed effects & interactions	parameter estimates ± s.e. for fixed effects		*P* value	*n* plants	*χ^2^*	variance estimate for random effect ± s.d.	statistical test
Latency until first visit	treatment (CTR)	CU	-0.33 ± 0.24	0.178	14	16.59	position of plants 0.02 ± 0.13	GLMM (Gamma, log link)
		FR	0.51 ± 0.23	**0.028**	18			
	cultivar (Darselect)	Malwina	0.02 ± 0.23	0.927	20	0.01		
	open flowers	open flow.	-0.10 ± 0.13	0.432	41	0.64		
Duration of first visit	treatment (CTR)	CU	0.19 ± 0.41	0.651	14	4.12	position of plants 0.16 ± 0.39	GLMM (Gamma, log link)
		FR	0.72 ± 0.39	0.068	18			
	cultivar (Darselect)	Malwina	-0.94 ± 0.31	**0.002**	20	9.35		
	open flowers	open flow.	-0.11 ± 0.26	0.668	41	0.18		
Visit frequency	treatment (CTR)	CU	-0.35 ± 0.07	**<0.001**	37	24.15	position of plants 0.006 ± 0.08 colony ID 0.072 ± 0.27	GLMM (poisson, log link)
		FR	-0.21 ± 0.07	**0.003**	38			
	cultivar (Darselect)	Malwina	0.18 ± 0.18	0.319	50	0.99		
	open flowers	open flow.	0.45 ± 0.08	**<0.001**	111	34.04		
	cultivar (Darselect) X open flowers	Malwina X open flow.	-0.34 ± 0.09	**<0.001**	50	13.30		
Visit duration	cultivar (Darselect)	Malwina	-0.41 ± 0.11	**0.007**	50	7.27	position of plants 0.004 ± 0.06	GLMM (Gamma, log link)
	open flowers	open flow.	0.17 ± 0.12	0.146	111	2.12		

Plants [Fragaria × ananassa var. Darselect (DS), Malwina (MW)], grown and offered in a greenhouse (2020) were untreated [control (CTR)] or fungicide-treated [Cuprozin^®^ progress (CU), SWITCH^®^ (FR)]. Tested were the latency until the first flower visit, duration of that first visit, frequency of plant visits and total duration of plant visits. Displayed are parameter estimates with std. errors and *P*-values indicating levels of significance obtained from generalised linear mixed effect models [GLMM] for the effects of treatment (CTR, CU, FR), cultivar (Darselect, Malwina), and number of open flowers. The reference level for multilevel fixed effects is given in brackets. The number of replicates is presented as well as the *χ^2^* value for fixed effects. Variance estimates with std. deviations are shown for the random effect position of plants and colony ID. Significant values (p < 0.05) are highlighted in bold.

Over the trial period of 15 min, the frequency of plant visits was significantly influenced by the interaction between cultivar and number of open flowers (Fig 4C, Table 2). The more flowers a Malwina plant had, the less it was visited (Fig 4C, Table 2). Besides that, flowers of CTR plants received significantly more visits than flowers of fungicide-treated plants (Fig 4C, Table 2). Additionally, the count of open flowers significantly impacted the overall visit frequency; the more flowers a plant had, the more often it was visited (Table 2). The total visit duration was on average, but not significantly, 1.5 times lower on flowers of Malwina plants than on flowers of the Darselect plants (Fig 4D, Table 2).

## Discussion

### Fungicide treatment alters floral volatile profiles and might affect nectar fungi

Our study revealed that the two fungicide treatments significantly impacted floral volatile profiles of strawberry plants in the greenhouse in 2020 (Fig 1B). This is in line with the first part of our first hypothesis that the treatments would affect the flower volatile profiles and additionally disclosed a significant effect on the volatile benzyl benzoate (Fig 1A). The reduced relative amount of emitted benzyl benzoate in the CU (Cuprozin^®^ progress) treatment is interesting, because it is an intermediate between benzoic acid and phenylalanine, which is an amino acid and precursor of different volatile compounds [11]. Due to heavy metal stress and the concomitant oxidative stress, phenylalanine could have been deaminated by the phenylalanine ammonia lyase, to support the plants photosynthetic efficiency instead of synthesising volatiles [11, 61]. In fruits [9] as well as in leaf material of strawberry plants, a higher phenylalanine content was found when plants were treated with the fungicide Cuprozin^®^ progress or SWITCH^®^ (FR treatment; A.-C. Voß, unpublished). In contrast, SWITCH^®^ had no significant effect on the benzyl benzoate content. Regarding the summarized content of emitted floral volatiles from field-grown strawberries in 2020, the summarized content of each treatment group relative to each other were similar to the relation pattern of the same groups in greenhouse-grown plants (S2A Fig). The lower amounts of volatiles detected in the field-sampled plants could be due to a higher air movement outside, which might have diluted the PDMS-trapped amounts, compared to the samples collected in the greenhouse. Additionally, field plants are exposed to elevated levels of environmental factors such as rain, heat, and UV-radiation, which could explain the difference to the greenhouse volatile profiles.

The floral bouquet does not only originate from the plant, including pollen volatiles [11, 62] but can also be modified by nectar microbes [14–16]. Differences found in the floral volatile profiles (Fig 1) between the strawberry plants of different fungicide treatments may be explained by the impacts on plant metabolism as well as on the nectar microbe composition. Indeed, we found slight differences in the fungi community composition in the nectar of our experimental plants placed in the field in 2021 (Fig 2C). However, the volatile and microbial field-samples were not taken in the same year, so we cannot directly test for such a link. Moreover, microbe samples were only taken from very few samples.

Nevertheless, the Shannon diversity was slightly higher in control flowers than in those of the FR-treated flowers (Fig 2B). Based on the results of the nectar analysis, it is not possible to make a final statement regarding the first hypothesis, that the fungicide treatment would affect the composition of fungal species in the nectar. However, the observed slight difference in microbe abundance (Fig 2C) could be explained by the fungicide formulation, as the fungicide SWITCH^®^ can potentially reduce fungal abundance in nectar, while Cuprozin^®^ progress is a bactericide and fungicide that could affect both fungi and bacteria. Around one third of the detected fungal species in this study belonged to the Basidiomycetes, which are frequently found on plant surfaces, belonging to the phylloplane rather than the nectar [63]. A possible explanation for their occurrence in our nectar samples could be the used nectar-washing method, where we introduced sugar-water to the whole flower cavity. Basidiomycetes were also isolated from the nectar of other Rosaceae species, which was attributed to the close arrangement of the flower parts [63]. The remaining species in our study belonged to the Ascomycetes, of which more than half belonged to the genus *Metschnikowia*. This genus is known to be common in nectar and found with a high occurrence [18, 63].

### Fungicide treatment did not affect pollen traits but the pollen viability

Regarding the pollen, we expected the fungicide treatment to lead to a lower pollen weight, pollen protein content and live-to-dead ratio. However, we could not observe such influences on the pollen weight or pollen protein content from plants in the greenhouse (2020 data, Table 1). In general, the protein content of our experimental plants was on average 0.7–3% and thus substantially lower than usual for strawberry plants, which may be related to the growth conditions. Depending on the cultivar, the percentage of the protein content in pollen can vary between 20–42% [64]. Nevertheless, we found an effect of the fungicide application on pollen traits: The live-to-dead ratio of pollen grains and total amount of pollen grains were significantly influenced by the fungicide treatment in plants in the field (2020 data, Fig 3, Table 1). Although CU-treated plants had a higher number of pollen grains, they contained significantly fewer viable grains, pointing to a lower fertility. The viability is also a proxy for the pollen protein content [19], which suggests that our CU-treated plants in the field are likely to have a lower protein content than untreated field plants. However, our pollen protein analysis from greenhouse plants (2020 data) was inconclusive as the protein content was considerably lower than typical for strawberry plants. In strawberry cultivars, pollen viability is important, as a high proportion of viable pollen can increase pollinator visits and thus prevent fruit deformation [19, 27].

### Bumblebee pollinating behaviour is affected by fungicide treatment

In the greenhouse experiment with bumblebees as pollinators (2020 data), our study revealed that flowers of FR-treated plants were visited significantly later than control or CU-treated flowers, which is partly consistent with our hypothesis (Fig 4, Table 2). Contrary to our expectations, the duration of the first visit was not shorter on fungicide-treated plants than on control plants. It is important to consider that the sample size for the latency until the first visit was small, and a higher number of replicates is required to further support these results. Although FR-treated flowers had a higher volatile emission than control flowers, the latency until the first visit was longer than in control plants, which might indicate that pollinators find these flowers less attractive. The longer latency to visit FR-treated plants might be due to potential fungicide-mediated changes in the microbial community. Nectar bacteria are known to have a higher emission of volatiles acting repellent on pollinators [17, 18, 65], compared to the more attractive emissions of nectar yeasts [14, 66]. In comparison with control flowers, no effect of the CU-treatment was observed for the latency until the first visit of bumblebees or the amount of floral volatiles. When comparing the trials of the two cultivars in terms of the duration of the first visit, flowers of Malwina plants seem to be less attractive in terms of taste or pollen quality than those of Darselect plants, as the first visit was shorter and bumble bees may directly searched for a higher quality flower.

The results of the visit frequency by bumblebees were surprising and contrary to our hypothesis, as we expected that the treatment effects on the visit frequency may vanish over the trial period. Yet, a clear difference between the less visited fungicide-treated flowers and the more often visited control flowers only became apparent over the whole length of the trials. Thus, the fungicide treatment reduced the attractiveness of the respective flowers and thereby the foraging behaviour of the bumblebees, which is in line with Tamburini et al. [67]. However, it is also important to note that we did not have any additional control with water sprayed plants which may have contributed to the difference between control and treated flowers, as the liquid or moisture from the treatment might also have affected on the flowers [68]. Nevertheless, our results support our hypothesis regarding the expected difference in the frequency and duration of plant visits between the two strawberry cultivars. The impact of the flowers on the overall visit frequency is noteworthy, as Darselect and Malwina flowers are similar in size and shape. Over all trials, Darselect plants had most often one open flower but were visited more often and more prolonged than Malwina plants with several open flowers. One reason for this difference might be a different amount of green leaf volatiles released by the two cultivars. The floral volatiles of Darselect plants were not measured in our study, but it is known that the amount of green leaf volatiles can differ between strawberry cultivars [12]. In strawberry flowers, these volatiles can have a repellent effect on bumblebees, influencing their foraging behaviour [12]. In the greenhouse trials (2020 data), bumblebees visited the fungicide-treated flowers significantly less often, compared to control flowers. However, this did not affect their overall visit duration. The lower visit frequency towards FR-treated flowers might be linked to a lower diversity in the fungal composition, as we found a potentially lower fungal diversity in FR-treated plants in the field (2021 data). Nectar microbes can alter the chemical properties of nectar, which bees use together with pollen taste to decide which flowers to visit [14, 19]. Previous research on fungicide-treated cranberries has shown that fungicides reduced foraged cranberry pollen and increased collected non-cranberry pollen by honeybees, depending on the specific fungicide used [69]. Another potential explanation for the lower visit frequency could be the direct effect of fungicides on altering the taste, as shown with sugar water [70], which in turn can influence the foraging behaviour of bees. Furthermore, the scent of the used fungicides could have interfered with the recognition of common floral scent [71].

In this study, actual strawberry plants were used and last sprayed a few days before the trials leading to more random, but also natural variation in terms of olfactory and visual traits, compared to artificial flowers or scent feeders, as used in several other studies on strawberry pollination (e.g., [15, 71]. Another critical aspect of fungicide use in crop cultivation that needs to be considered is that the fungicide treatment of the plants may affect not only the foraging behaviour of bumblebees but also the physiology of the insects. When bumblebees are exposed to field-relevant amounts of fungicides, this can lead to fewer workers and to the production of smaller queens in the colonies [6]. For example, fludioxonil, which is formulated in the fungicide SWITCH^®^, can cause disruption of mitochondrial respiration in the flight muscles of bumblebees [72]. Copper-based fungicides have also been shown to increase mortality in honeybees even at concentrations recommended by the manufacturer [73]. However, the physiological response of the insects may not only depend on the used active ingredient but also on the co‑formulant in the fungicide product i.e., alcohol ethoxylates [74] as well as on the particular crop species that was treated [75]. In future experiments, it would be important to analyse whether, apart from volatile changes in the flower, the bumblebees also recognise the fungicides themselves, e.g., by the antennae, the tarsi or their glossae. In addition, it would be interesting to investigate the influence of fungicide treatment on the visual perception of the flowers.

## Conclusions

In conclusion, our results indicate that under semi-controlled conditions, fungicide treatments can affect flower attributes as well as the attraction and visit frequency of flowers to bumblebees, even at relatively low application rates. Plant-pollinator interactions are complex, with many factors such as volatiles, pollen, and nectar quality, as well as visual cues playing important roles for successful pollination. Although fungicide treatments can activate a defence response in plants, they influence the plant metabolism, including pollen and eventually nectar microbes. This can impact the viability and protein content of pollen and thus pollen dispersal by pollinators, as a high protein content attracts pollinating insects. Attracted pollinators are effective in pollinating flowers, preventing the deformation of strawberry fruits.

## Supporting information

S1 Method Strawberry field design. (PDF)

S1 Fig Outline of the strawberry field. (PDF)

S2 Fig Flower volatiles collected from strawberry plants in greenhouse and field. (PDF)

S1 Table Overview of the conducted experiments in this study. (PDF)

S2 Table Flower volatile compounds with their details. (PDF)

S3 Table Output of (generalised) linear models for volatiles in the greenhouse. (PDF)

S4 Table Output of (generalised) linear models for volatiles in the field. (PDF)

S1 File README file. (PDF)

S1 Dataset (XLSX)

S1 Text R script. (R)
