# Supplementary material for: Fungicides and strawberry pollination–Effects on floral scent, pollen attributes and bumblebee behavior
Source: PLoS One. 2023 Jul 27;18(7):e0289283. doi: 10.1371/journal.pone.0289283 (PMC10374001; doi:10.1371/journal.pone.0289283)
Supplement: S1 Method — (PDF) [file pone.0289283.s001.pdf]

S1 Method. Strawberry field design

Strawberry plants (Kraege Beerenpflanzen GmbH & Co.KG, Telgte, Germany) were planted to a field site close to Bielefeld University (latitude: 52.033684, longitude: 8.495052; 146 m a. s. l.) in June 2019. The field margins were 4 by 5 m and were divided into four blocks with three plots (S1 Fig). Untreated strawberry plants of the two *Fragaria* × *ananassa* cultivars, Darselect and Malwina, as well as the wild strawberry *Fragaria vesca* were planted around each block and between the plots. The three plant types were equally distributed over the field and planted with 25 cm distance between each plant. These plants were in the field throughout the years and the experimental plants were later placed individually in the rows (coloured rectangles in the Figure) for the field observations. After the fungicide treatments in the greenhouse, the experimental plants were transferred to the field site and placed in the assigned plots for two weeks.
