# Supplementary material for: Fungicides and strawberry pollination–Effects on floral scent, pollen attributes and bumblebee behavior
Source: PLoS One. 2023 Jul 27;18(7):e0289283. doi: 10.1371/journal.pone.0289283 (PMC10374001; doi:10.1371/journal.pone.0289283)
Supplement: S1 Fig — (PDF) [file pone.0289283.s002.pdf]

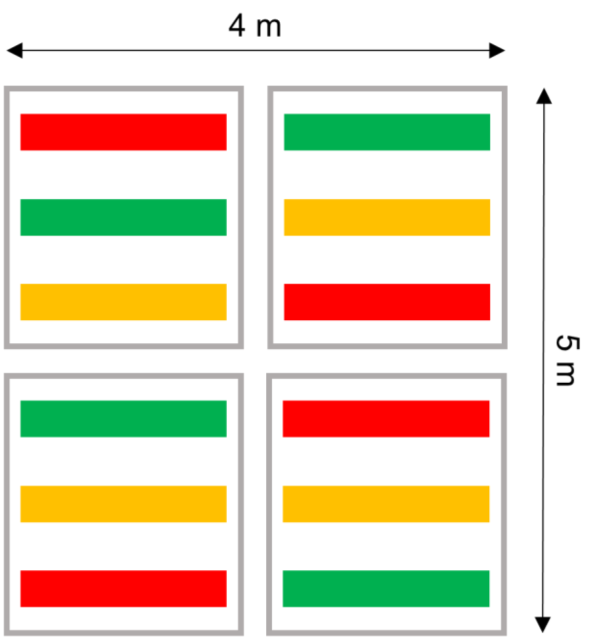

S1 Fig. Outline of the strawberry field.
Displayed are the margin of the field (black arrows), the blocks (grey rectangle) and plots (rectangles; green: control, yellow: Cuprozin® progress, red: SWITCH®). Permanently planted strawberry (*Fragaria* × *ananassa*) plants were around each plot and the five experimental plants were placed in the respective plot.
