# Supplementary material for: Fungicides and strawberry pollination–Effects on floral scent, pollen attributes and bumblebee behavior
Source: PLoS One. 2023 Jul 27;18(7):e0289283. doi: 10.1371/journal.pone.0289283 (PMC10374001; doi:10.1371/journal.pone.0289283)
Supplement: S2 Fig — (PDF) [file pone.0289283.s003.pdf]

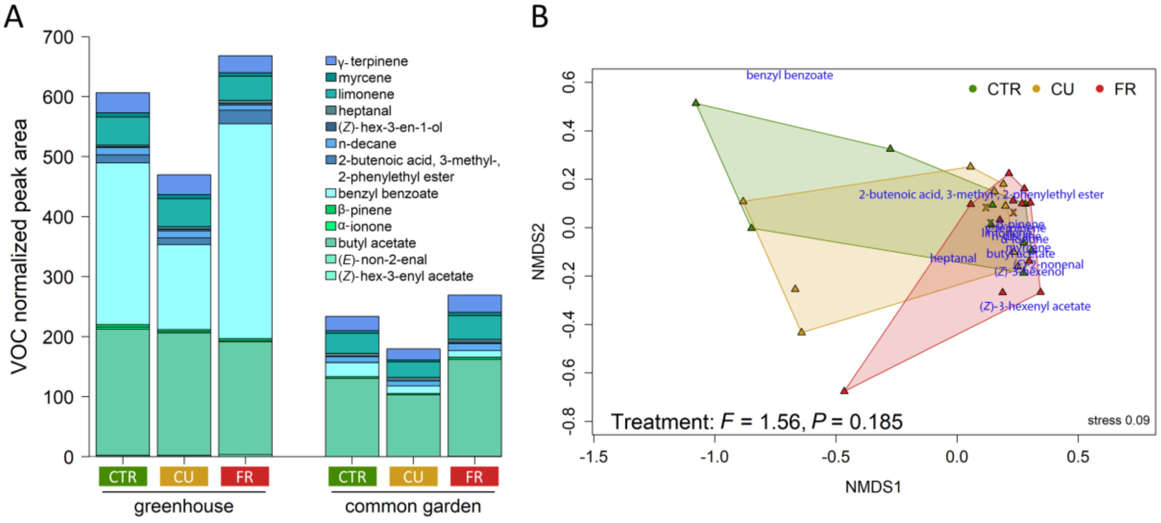

S2 Fig. Flower volatiles collected from strawberry plants in greenhouse and field.

Flower volatiles collected from strawberry plants of the cultivar Malwina (*Fragaria* × *ananassa*) grown in the greenhouse (**A**, left) and placed in the field (**A**, right side and **B**) in 2020, which were treated with fungicides [control (CTR), Cuprozin® progress (CU), SWITCH® (FR)]. (**A**) Volatile composition (averaged over replicates within groups). (**B**) Non-metric multidimensional scaling (NMDS; with Kulczinsnky distance matrix) of the volatile composition with scores (coloured symbols; samples within each group are surrounded by convex hulls and the corresponding medians of the groups shown as crosses) and loadings (blue compound names). Results of ADONIS are shown in the graph (**B**); n = 7-11 replicates per fungicide treatment.
