# Supplementary material for: Fungicides and strawberry pollination–Effects on floral scent, pollen attributes and bumblebee behavior
Source: PLoS One. 2023 Jul 27;18(7):e0289283. doi: 10.1371/journal.pone.0289283 (PMC10374001; doi:10.1371/journal.pone.0289283)
Supplement: S1 Table — (PDF) [file pone.0289283.s004.pdf]

S1 Table. Overview of the conducted experiments in this study.

Experiment	Year	Environment	Used cultivar	Number of plants per treatment	Performed analyses	Used model
1	2020	Greenhouse	Malwina	07 - 08	Floral volatiles	(G)LMs (gaussian, link: identity or log; Gamma, link: inverse); NMDS with ADONIS (Kulczynski distance)
	2020	Field	Malwina	09 - 11	Floral volatiles	(G)LMs (gaussian, link: identity or log; Gamma, link: inverse); NMDS with ADONIS (Kulczynski distance)
2	2021	Field	Malwina	02 - 03	Nectar fungi	Shannon diversity
3	2020	Field	Malwina	10 - 12	Total number of pollen grains	GLM (poisson, link: log) with Tukey *post-hoc* test
	2020	Field	Malwina	10 - 12	Live-to-dead ratio of pollen grains	GLM (Gamma, link: log) with Tukey *post-hoc* test
4	2020	Greenhouse	Malwina	09 - 10	Pollen protein	GLM (Gamma, link: inverse) with Tukey *post-hoc* test
	2020	Greenhouse	Malwina	09 - 10	Pollen dw	GLM (Gamma, link: inverse) with Tukey *post-hoc* test
5	2020	Greenhouse	Malwina, Darselect	19 - 22	Bumblebee trials	GLMMs (Gamma or poisson, log link)

Overview of the experiments carried out in this study with their associated details such as year (2020, 2021), environment (greenhouse, field), used cultivar (*Fragaria* × *ananassa* var. Malwina, Darselect), number of plants, performed analyses and used statistical model.
