# Supplementary material for: Fungicides and strawberry pollination–Effects on floral scent, pollen attributes and bumblebee behavior
Source: PLoS One. 2023 Jul 27;18(7):e0289283. doi: 10.1371/journal.pone.0289283 (PMC10374001; doi:10.1371/journal.pone.0289283)
Supplement: S2 Table — (PDF) [file pone.0289283.s005.pdf]

S2 Table. Flower volatile compounds with their details.

Group	Compound	*m*/*z*	Rt [min]	RI
Alcohols	(*Z*)-3-hexenol	41	6.147	853
Aldehyde	(*E*)-2-nonenal	43	12.333	1165
	heptanal	57	7.28	905
Alkane	n-decane	57	9.36	999
Esters	(*Z*)-3-hexenyl acetate	43	9.5	1007
	butyl acetate	43	5.06	804
	benzyl benzoate	105	20.427	1785
	2-butenoic acid, 3-methyl-, 2-phenylethyl ester	104	17.047	1495
Terpenes	β-pinene	93	9.093	984
	limonene	68	10	1033
	γ-terpinene	93	10.55	1062
	α-ionone	121	16.207	1431
	myrcene	93	9.167	991

Flower volatile compounds of the strawberry cultivar Malwina (*Fragaria* × *ananassa*) with their mass-to-charge ratio (*m*/*z*), retention times (Rt) and retention index (RI).
