# Supplementary material for: Fungicides and strawberry pollination–Effects on floral scent, pollen attributes and bumblebee behavior
Source: PLoS One. 2023 Jul 27;18(7):e0289283. doi: 10.1371/journal.pone.0289283 (PMC10374001; doi:10.1371/journal.pone.0289283)
Supplement: S3 Table — (PDF) [file pone.0289283.s006.pdf]

S3 Table. Output of (generalised) linear models for volatiles in the greenhouse.

**Compound**	**Model**	***LRT***	***P***
(Z)-3-hexenol	LM (gaussian, log link)	1.68	0.430
(E)-2-nonenal	GLM (Gamma, inverse link)	0.72	0.699
heptanal	GLM (Gamma, inverse link)	1.14	0.566
n-decane	LM (gaussian, identity link)	1.84	0.398
(Z)-3-hexenyl acetate	GLM (Gamma, inverse link)	0.71	0.700
butyl acetate	GLM (Gamma, inverse link)	0.27	0.874
benzyl benzoate	GLM (Gamma, inverse link)	15.26	**<0.001**
2-butenoic acid, 3-methyl-, 2-phenylethyl ester	LM (gaussian, identity link)	8.23	**0.016**
β-pinene	GLM (Gamma, inverse link)	0.69	0.707
limonene	GLM (Gamma, inverse link)	0.66	0.718
γ-terpinene	GLM (Gamma, inverse link)	0.66	0.720
α-ionone	GLM (Gamma, inverse link)	1.71	0.425
myrcene	GLM (Gamma, inverse link)	0.54	0.764
total content	GLM (Gamma, inverse link)	4.77	0.092

Output of (generalised) linear models [(G)LM] for individual volatile compounds (normalized peak area) of flowers of the strawberry cultivar Malwina (*Fragaria* × *ananassa*) grown in the greenhouse (2020) with the factor treatment (CTR, CU, FR). In case of zero values in the dataset a value of $1e^{-07}$ was added. Significant *P*-values ($P < 0.05$) are highlighted in bold.
