# Supplementary material for: Fungicides and strawberry pollination–Effects on floral scent, pollen attributes and bumblebee behavior
Source: PLoS One. 2023 Jul 27;18(7):e0289283. doi: 10.1371/journal.pone.0289283 (PMC10374001; doi:10.1371/journal.pone.0289283)
Supplement: S4 Table — (PDF) [file pone.0289283.s007.pdf]

S4 Table. Output of (generalised) linear models for volatiles in the field.

**Compound**	**Model**	***LRT***	***P***
(Z)-3-hexenol	LM (gaussian, log link)	4.30	0.116
(E)-2-nonenal	GLM (Gamma, inverse link)	0.92	0.633
heptanal	GLM (gamma, inverse link)	0.5	0.780
n-decane	LM (gaussian, identity link)	2.25	0.325
(Z)-3-hexenyl acetate	GLM (Gamma, inverse link)	2.75	0.253
butyl acetate	GLM (Gamma, inverse link)	1.75	0.416
benzyl benzoate	GLM (Gamma, inverse link)	3.23	0.199
2-butenoic acid, 3-methyl-, 2-phenylethyl ester	GLM (Gamma, inverse link)	-	-
β-pinene	GLM (Gamma, inverse link)	1.61	0.448
limonene	GLM (Gamma, inverse link)	2.15	0.341
γ-terpinene	GLM (Gamma, inverse link)	2.31	0.315
α-ionone	GLM (Gamma, inverse link)	-	-
myrcene	GLM (Gamma, inverse link)	1.97	0.374
total content	GLM (Gamma, inverse link)	1.93	0.381

Output of (generalised) linear models [(G)LM] for the individual volatile compounds (normalized peak area) of flower from the strawberry cultivar Malwina (*Fragaria* × *ananassa*) from the field (2020) with the factor treatment (CTR, CU, FR). In case of zero values in the dataset a value of $1e^{-07}$ was added.
