# Supplementary material for: Fungicides and strawberry pollination–Effects on floral scent, pollen attributes and bumblebee behavior
Source: PLoS One. 2023 Jul 27;18(7):e0289283. doi: 10.1371/journal.pone.0289283 (PMC10374001; doi:10.1371/journal.pone.0289283)
Supplement: S1 File — (PDF) [file pone.0289283.s008.pdf]

This readme file was generated by Ann-Cathrin Voß

DATA ASSOCIATED WITH THE PUBLICATION

Fungicides and strawberry pollination – effects on floral scent, pollen attributes and bumblebee behaviour

Ann-Cathrin Voß[1*], Madeleine Hauertmann[1], Michelle-Celine Laufer[1], Alexander Lach[2], Robert R. Junker[2], Elisabeth J. Eilers[1,#a]

[1]Department of Chemical Ecology, Bielefeld University, Universitätsstr. 25, 33615 Bielefeld, Germany

[2]Evolutionary Ecology of Plants, Philipps-University Marburg, Karl-von-Frisch-Str. 8, 35043 Marburg, Germany

[#a]CTL GmbH Bielefeld, Krackser Straße 12, 33659 Bielefeld, Germany

*Correspondence: a.voss@uni-bielefeld.de (ACV)

Author Contact Information

Corresponding Author

Ann-Cathrin Voß – Department of Chemical Ecology, Bielefeld University, Universitätsstr. 25, 33615 Bielefeld, Germany; ORCID: https://orcid.org/0000-0003-3269-9912; Email: a.voss@uni-bielefeld.de

Authors

Madeleine Hauertmann – Department of Chemical Ecology, Bielefeld University, Universitätsstr. 25, 33615 Bielefeld, Germany

Michelle-Celine Laufer – Department of Chemical Ecology, Bielefeld University, Universitätsstr. 25, 33615 Bielefeld, Germany

Alexander Lach – Evolutionary Ecology of Plants, Philipps-University Marburg, Karl-von-Frisch-Str. 8, 35043 Marburg, Germany

Robert R. Junker – Evolutionary Ecology of Plants, Philipps-University Marburg, Karl-von-Frisch-Str. 8, 35043 Marburg, Germany; ORCID: https://orcid.org/0000-0002-7919-9678

Elisabeth J. Eilers – Former affiliation: Department of Chemical Ecology, Bielefeld University, Universitätsstr. 25, 33615 Bielefeld, Germany; Present affiliation: CTL GmbH Bielefeld, Krackser Straße 12, 33659 Bielefeld, Germany; ORCID: https://orcid.org/0000-0002-2159-7788

Supplementary Data and Analysis

Figures, tables, data, R script, and analyses of floral scent, pollen attributes and bumblebee behaviour responses to fungicide treatment in greenhouse and field experiments in Bielefeld, Germany in 2021 and 2022.

Experiment description

Two cultivars of the strawberry (*Fragaria × ananassa*, Rosaceae) were either kept untreated (control) or treated with either Cuprozin® progress (for organic cultivation) or SWITCH® (for conventional cultivation) fungicide. We analysed various flower traits including volatiles, pollen weight, pollen protein, and the attraction of bumblebees towards the flowers in the greenhouse. Additionally, we analysed the viability of pollen and pollen live-to-dead ratio, as well as the composition of nectar fungi in the field.

DATA-SPECIFIC INFORMATION

Details for: Dataset_Manuscript_Voss_et_al.xlsx

* Description: a comma-delimited file containing all chemical data in separate sheets.

* Format(s): .xlsx

* Size(s): 103 KB

Table of Contents

* README
* volatiles_greenhouse_field
* flower_diameter
* nectar_fungi_taxon
* nectar_yeast_diversity
* pollen_dw_protein
* pollen_alive_ratio
* bumblebee_1.visit
* bumblebee_overall.visits

Sheet/Column Details

Sheet / Column	explanation
**README**	column details of each sheet (see below)
**volatiles_greenhouse_field**	
GH_field	plant placement; greenhouse: GH; field: FIE
type	strawberry cultivar; Darselect: DS; Malwina: MW
treatment	fungicide treatment; no application: CTR; Cuprozin® progress: CU; SWITCH®: FR
id	individual identification number
(*Z*)-3-hexenol	(*Z*)-3-hexenol content in the floral headspace [normalized peak area]
(*E*)-2-nonenal	(*E*)-2-nonenal content in the floral headspace [normalized peak area]
heptanal	heptanal content in the floral headspace [normalized peak area]
n-decane	n-decane content in the floral headspace [normalized peak area]
(*Z*)-3-hexenyl acetate	(*Z*)-3-hexenyl acetate content in the floral headspace [normalized peak area]
butyl acetate	butyl acetate content in the floral headspace [normalized peak area]
benzyl benzoate	benzyl benzoate content in the floral headspace [normalized peak area]
2-butenoic acid, 3-methyl-, 2-phenylethyl ester	2-butenoic acid, 3-methyl-, 2-phenylethyl ester content in the floral headspace [normalized peak area]
b-pinene	b-pinene content in the floral headspace [normalized peak area]
limonene	limonene content in the floral headspace [normalized peak area]
g-terpinene	g-terpinene content in the floral headspace [normalized peak area]
a-ionone	a-ionone content in the floral headspace [normalized peak area]
myrcene	myrcene content in the floral headspace [normalized peak area]
total.voc	total sum of volatile content in the floral headspace [normalized peak area]
**flower_diameter**	
type	strawberry cultivar; Darselect: DS; Malwina: MW
treatment	fungicide treatment; no application: CTR; Cuprozin® progress: CU; SWITCH®: FR
id	individual identification number
total_flower_mm	total floral diameter of volatile sampled flowers [mm]
receptacle_mm	receptacle diameter of volatile sampled flowers [mm]
**nectar_fungi_taxon**	
ASV_ID	amplicon sequence variant (ASV) identification number
confidence	confidence of the conformity
domain	domain of fungi in nectar
phylum	phylum of fungi in nectar

class	class of fungi in nectar
order	order of fungi in nectar
family	family of fungi in nectar
genus	genus of fungi in nectar
species	species of fungi in nectar
**nectar_yeast_diversity**	
GH_field	plant placement; greenhouse: GH; field: FIE
type	strawberry cultivar; Darselect: DS; Malwina: MW
treatment	fungicide treatment; no application: CTR; Cuprozin® progress: CU; SWITCH®: FR
id	individual identification number
sample_id	individual sample identification number
collection_timestamp	collection day and time of sampling
description	description of the sample material
elevation	height of land above sea level at the field site [m]
env_biome	description of the location
env_feature	description of the specific biome
env_material	description of the sampled material
env_package	description of the sample environment
geo_loc_name	geographical origin of the field side
host_scientific_name	scientific name of the host
latitude	description of the latitude of the field site [DD]
longitude	description of the longitude of the field site [DD]
qiita_study_id	qiita study identification number
sample_type	description of the sample material
taxon_id	taxon identification number of *Fragaria* **×** *ananassa*
title	title of the qiita study
hereinafter all ASVs	**column Y to NJ** all found amplicon sequence variants (ASVs)
**pollen_dw_protein**	
GH_field	plant placement; greenhouse: GH; field: FIE
type	strawberry cultivar; Darselect: DS; Malwina: MW
treatment	fungicide treatment; no application: CTR; Cuprozin® progress: CU; SWITCH®: FR
id	individual identification number
dw_mg	pollen dry weight per flower and per plant [mg]
protein_content	pollen protein content [%]
**pollen_alive_ratio**	
GH_field	plant placement; greenhouse: GH; field: FIE
type	strawberry cultivar; Darselect: DS; Malwina: MW
treatment	fungicide treatment; no application: CTR; Cuprozin® progress: CU; SWITCH®: FR
id	individual identification number
pollen_alive	number of viable pollen per sample
total_pollen	total number of pollen per sample
live-to-dead-ratio	ratio of viable to dead pollen per sample

bumblebee_1.visit	
GH_field	plant placement; greenhouse: GH; field: FIE
type	strawberry cultivar; Darselect: DS; Malwina: MW
treatment	fungicide treatment; no application: CTR; Cuprozin® progress: CU; SWITCH®: FR
id	individual identification number
group	combination of type and treatment
trial	trial number
pot_position_1.visit	plant pot position on the orange rectangle during the trials
latency_1.visit	latency until the first visit of a flower [s]
duration_1.visit	duration of the first visit of a flower [s]
flower_open_1.visit	number of open flowers per plant
beecolony_1.visit	ID of the three colonies

bumblebee_overall.visits	
GH_field	plant placement; greenhouse: GH; field: FIE
type	strawberry cultivar; Darselect: DS; Malwina: MW
treatment	fungicide treatment; no application: CTR; Cuprozin® progress: CU; SWITCH®: FR
id	individual identification number
group	combination of type and treatment
trial	trial number
pot_position_overall	plant pot position on the orange rectangle during the trials
overall.visits_freq	overall visit frequency during the trial [s]
overall.visits_duration	overall visit duration during the trial [s]
overall.visits_duration_mean	mean of the duration per visit during the trial [s]
overall.visits_duration_std_dv	standard deviation of the duration per visit during the trial [s]
flower_open_overall	number of open flowers per plant
fruit_ripe_overall	number of ripe fruits per plant
beecolony_overall	ID of the three colonies

CODE / SOFTWARE

Details for: R script_Manuscript_Voss_et_al.R

* Description: R script file for the analysis of all metabolite calculations.

* Format(s): .R

* Size(s): 24.5 KB

Table of Contents within the file

* All calculations for total greenhouse volatiles (greenhouse, 2020)

* All calculations for flower diameter of volatile sampled flowers (greenhouse, 2020)

* All calculations for the nectar yeasts (field, 2021)

* Calculation for the pollen dry weight and protein (greenhouse, 2020)

* Calculation for total pollen count, viable pollen and live-to-dead-ratio (field, 2020)

* All calculations for the bumblebee trials (greenhouse, 2020)

* Supplement

    All calculations for total field volatiles (field, 2020)

    Calculations of individual greenhouse volatiles (greenhouse, 2020)

    Calculations of individual field volatiles (field, 2020)

Note

Data.csv files must be placed in your working directory for the analyses to work.

Funding information

This work was funded by the Scholarship program of the German Federal Environmental Foundation (Deutsche Bundesstiftung Umwelt - DBU). We acknowledge support for the publication costs by the Open Access Publication Fund of Bielefeld University and the Deutsche Forschungsgemeinschaft (DFG).
